# Effect of Different Watering Regimes on Olive Oil Quality and Composition of Coratina Cultivar Olives Grown on Karst Soil in Croatia

**DOI:** 10.3390/foods11121767

**Published:** 2022-06-15

**Authors:** Karolina Brkić Bubola, Šimun Kolega, Šime Marcelić, Zoran Šikić, Ana Gašparović Pinto, Marko Zorica, Dora Klisović, Anja Novoselić, Maja Jukić Špika, Tomislav Kos

**Affiliations:** 1 Department of Agriculture and Nutrition, Institute of Agriculture and Tourism, Karla Huguesa 8, HR-52440 Poreč, Croatia; karolina@iptpo.hr (K.B.B.); dora@iptpo.hr (D.K.); anovoselic@iptpo.hr (A.N.); 2 Department of Ecology, Agronomy and Aquaculture, University of Zadar, Square of Prince Višeslav 9, HR-23000 Zadar, Croatia; simemarcelic@unizd.hr (Š.M.); zsikic@unizd.hr (Z.Š.); agasparov@unizd.hr (A.G.P.); mzorica@unizd.hr (M.Z.); tkos@unizd.hr (T.K.); 3 Department of Applied Sciences, Institute for Adriatic Crops and Karst Reclamation, Via Duilova 11, HR-21000 Split, Croatia; Maja.Jukic.Spika@krs.hr; 4 Centre of Excellence for Biodiversity and Molecular Plant Breeding (CoE CroP-BioDiv), Svetošimunska Cesta 25, HR-10000 Zagreb, Croatia

**Keywords:** watering regimes, *Olea europea* L., olive oil, quality, volatile compounds, phenolic compounds, fatty acids, sensorial characteristics

## Abstract

Croatian islands are olive growing areas characterized by poor conditions for olive trees because of karst soil and a precipitation deficiency. Under these conditions, irrigation is a very important factor for constant olive oil production. This paper aims to investigate the effects of different watering regimes on quantity, sensory and chemical quality and composition of Coratina cv. olive oil obtained from trees grown on ameliorate karst soil during two harvesting years. Olive trees were subjected to rainfed conditions and three different irrigation treatments (T_1_—deficit irrigation representing the usual producer’s practice, T_2_—regulated deficit irrigation in respect to phenological stages, T_3_—full irrigation). Irrigation treatments increased oil yield compared to rainfed conditions (T_1_ + 58%, T_2_ + 66% and T_3_ + 74%, representing average values for both studied years). All olive oil samples were of extra quality. Irrigation led to a decrease in carotenoids, volatiles, polyunsaturated fatty acids and linolenic acid contents, with no difference found among irrigation treatments. Total phenols and secoiridoids concentration was not affected by irrigation, indicating that similar oil quality could be achieved with less demand on the water supply. Obtained results could help producers to define a suitable irrigation management in particular conditions of ameliorate karst.

## 1. Introduction

Olive (*Olea europea* L.) is an evergreen fruit tree [1], well adjusted to Mediterranean climate conditions, that is characterized by short, cold and wet winters and long, dry and hot summers [2]. Worldwide, more than 10 million ha of olives are grown, but 90% of olive cultivation is localized in the Mediterranean Basin [3].

Olive cultivation has a long tradition in Croatia. The olive tree is one of the most economically important crops in the Adriatic region of Croatia and olive orchards cover around 20,000 ha of land; the production of olives is about 33,000 t per year [4]. Olive growing regions in Croatia cover 1000 km of the coast line and include numerous islands, divided in six sub-regions which include the Regions of the Istrian peninsula, Kvarner and Hrvatsko Primorje, Northern Dalmatia, Central Dalmatia, South Dalmatia and Dalmatian Hinterland [5]. All Croatian olive growing regions are located in areas with Mediterranean–type climates, but Croatian islands are especially characterized as having demanding conditions for growing olive trees because of the types of soil (karst) and lack of water for irrigation.

It is predicted that climate changes will have an effect on tree growth, which could in turn have a negative effect on the production of olive oil [6,7,8]. Climate change, characterized by lower precipitation and higher temperatures, could prove a challenge for traditional olive growing practices in Mediterranean countries [7,9] including Croatia. Long term drought periods during intensive drupe growth, strong stormy winds causing erosion of formerly shallow soils and alterations in phenological phases are already affecting oil yield [10]. Over the past decades, analysis of data from the meteorological station Biograd na moru shows that this area has seen a temperature increase of 0.5 °C per decade and a 3% reduction in precipitation [11]. In general, in Croatia olive trees are cultivated under rainfed conditions in traditional orchards characterized by low plantation density and low olive fruit production due to thermal and water stress during the growing season, especially during the warm and dry summer months (from the data of nearest CMHS meteo station, average total precipitation in June-July-August is around 90 mm) [12]. Since fruit yield, and consequently olive oil yield, depend on water availability [7,13,14], irrigation of olive trees under low rainfed conditions is a very important factor for olive oil production.

Irrigation of olive trees could be one of the possible adaptive measures that alleviates the detrimental impact of climate changes on olive yield [7]. There is evidence in the literature that irrigation management in olive orchards could increase olive and olive oil production [15]. Despite good resistance of olive trees to the water deficit, it is proven that there are several beneficial effects of irrigation, including an increase in fruit size, the fruit yield and oil content in the fruit of high-density Frantoio cv. olive orchards [16]. Olive oil is highly appreciated due to its high nutritional value and oxidative stability, related to the specific chemical composition, monounsaturated fatty acids and bioactive compounds such as phenolic compounds [15]. Olive oil quality and composition can be influenced by several factors, and one of them is irrigation [9]. Most of the authors agree on the negligible or no effect of irrigation on the basic olive oil quality parameters, such as free fatty acids, peroxide value and absorbance in the ultraviolet region as well as on the fatty acid composition [9,15,16]. Among chemical components of olive oil, phenolic compounds are most influenced by the amount of water applied to the olive trees and therefore the most investigated regarding the influence of irrigation [9,15,16]. In general, concentration of VOO phenolic compounds decreases as water availability in the soil increases, but inconsistent effects of irrigation on the olive oil phenolic compounds, as well as on volatile compounds, are found in the published literature [9,15,16,17,18], probably due to very complex response of olive oil composition to irrigation which also depends on cultivar, growing seasons as well as environmental conditions. This highlights the importance of long-term studies on these issues.

There is only a small percentage of intensive production olive orchards in Croatia [5] and irrigation systems are commonly implemented in such orchards as a strategy to increase quality and yield of olive oils. Due to limited water resources on the Croatian islands, a smart choice in olive tree watering regimes (for example, deficit irrigation) is of importance. Deficit irrigation strategies are based on supplying irrigation quantities below the irrigation needs for the maximum potential crop evapotranspiration (ET_c_), but with minimized impact of irrigation on crop performance [18]. Defined application of a deficit irrigation strategy in olive orchards is needed not only to improve oil yield, but to also obtain high quality olive oil. Moreover, olive growers experience difficulties in determining irrigation outbreak, water quantity and application rate which frequently results in overdue or unbalanced irrigation. This, on the other hand, can lead to unnecessarily reduced yield as well as increased production costs and negative environmental effects.

Current technologies in olive cultivation, as well in other crop production, increasingly rely on the “smart agriculture” concept. Introducing smart agriculture technologies and using autonomous support systems can, on the one hand, compensate for a lack of workforce, while advanced sensor-based technology can, on the other hand, enable precise and automatic water rate application, thus increasing water usage efficiency as well as the yield and the quality of the crop. Additionally, with the help of artificial intelligence (AI), algorithms can be used to filter the data directly derived from measuring various factors related to the plant, soil or environment, which will help the producer to make informed farm management decisions [19]. Under the Smart Agriculture Network (SAN) project, an AI model has been developed; a simple application tool with the aim of helping producers to make decisions related to the irrigation run time and water rate was tested for the first time in this study [20]. This ‘Internet of things’ (IoT) tool was developed to determine irrigation rate using the standard rate calculation method and involved irrigation based on plant phenological stage. Taking into consideration the main phenological stages’ water requirements, 80% of the total water requirement was applied before flowering, fruit growth and oil accumulation, while 50% of the required water was applied between these phenological stages. The goal was to provide enough water in critical growth and development stages, while reducing the total volume of added water in the conditions of limited water supply.

In light of the above, the aim of the present study was to investigate the influence of four different watering regimes applied during two years on the Coratina cv. olive trees grown on karst soils on the Croatian island, Dugi otok, with respect to the quality, composition and quantity of the obtained olive oil. Investigated watering regimes were rainfed conditions, deficit irrigation that represents the usual producer’s practice, deficit irrigation acquired by SAN technology in respect to phenological stages, and irrigation with 100% of ET_c_ level. Island Dugi otok is known for excessive dryness with elevated ET values [21]. To date, there is a lack of data regarding the influence of irrigation on Coratina cv. olive trees grown on karst soil in Croatia. Here, we discuss how different irrigation treatments will affect the main quality parameters used in the categorization of virgin olive oil. In addition, we report on how the amount of water applied to the studied soil type affects the composition of virgin olive oil (fatty acid, volatile composition, phenolic profile, pigments), which is used to distinguish olive from other vegetable oils. Finally, we tested for the first time, a new application tool developed by the SAN project that could help producers to make informed decisions related to irrigation run time and water use with respect to phenological stages.

## 2. Materials and Methods

### 2.1. Plant Material and Growing Area Characteristics

The study was conducted over two consecutive years in an olive grove on the island Dugi otok, near the location of Žman (43°57′42.95″ N, 15°7′21.53″ E). The average age of olive trees sampled was 10 years. The trees were planted as part of an expanded extensive olive orchard and the polyconic vase training system, up to 4 primary branches, was in use.

The local climate is Mediterranean, characterised by mild, rainy and moderately windy winters, with hot and dry summers [22]. Based on the data collected by the PinovaMeteo meteorological station, located inside the olive orchard grove, total rainfall in 2019 was 1037.9 mm, and 804 mm in 2020 [23].

**Figure 1:**
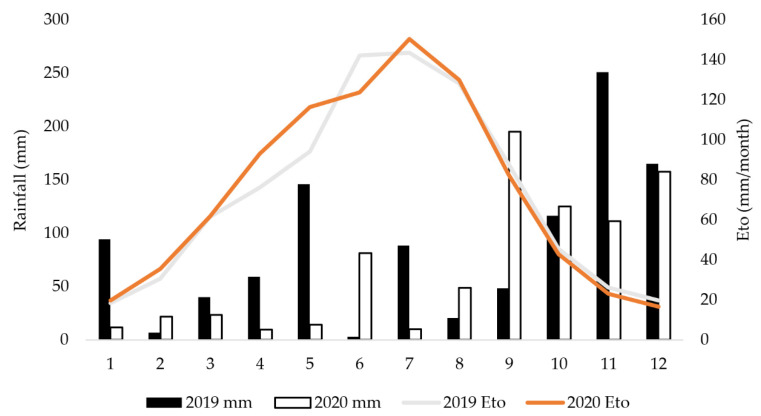
Monthly rainfall and monthly reference evapotranspiration in 2019 and 2020, from Žman, Dugi otok. ETo—reference evapotranspiration (obtained from the PinovaMeteo meteorological station [23]).

Figure 1 shows data on rainfall and reference evapotranspiration (ETo) during the two-year study period.

In 2019 there was more precipitation at the beginning of the vegetation and during the flowering period, while in 2020, rainfall was higher during the ripening period [23]. The average daily ETo, calculated based on the data obtained from the PinovaMeteo meteorological station, was 3.79 mm in 2020 during the irrigation season (April–October), while it was somewhat lower in 2019 (3.66 mm) [23].

The water used for irrigation in all three treatments was desalinated water from the producer’s water well situated in the olive grove. The water was analysed in the middle of the irrigation cycle during both study years (21 August 2019 and 12 August 2020). The main measured values were: pH (at 25 °C) 7.0 in 2019 and 7.9 in 2020; electroconductivity of 346 µS/cm in 2019 and 394 µS/cm in 2020; and total chlorides of 71.3 mg Cl/l in 2019 and 149 mg Cl/l in 2020. The values were higher during dry months, but the water was found to be adequate for irrigation throughout the irrigation cycle in both observed years [24,25].

The soil in the olive grove is characterized as ameliorate karst soils, with an anthropogenic horizon up to 40 cm in depth. The top layer of the soil profile is a silty clay texture with a skeletal content of 35.6%, while the deeper layer consists of clay and loam texture with a skeletal content of 61.8%. Average bulk soil density is 1.23 g/cm^3^. The average field water capacity, after correction for the skeletal content, was very low and estimated at 17.8%, while the wilting point was 10.2%. Soil pH was basic (7.9), rich in organic matter, well saturated with nitrogen (0.18 N), rich in potassium (31.3 mg K_2_O/100 g), and poor in phosphorus (5.84 mg P_2_O_5_/100 g) [26]. The grove is grass covered and regularly trimmed with cultivation of the surface area under the olive tree canopy.

The two-year experiment was conducted on 12 own-rooted trees of the Italian cultivar ‘Coratina’ highly adaptable to various environmental and cultivation conditions and characterised by a high and regular production. The variety bears large fruits of high variability in size and the oil yield has a very fruity flavour and high concentration of polyphenolics [27,28,29].

### 2.2. Watering Regimes

The experiment involved four watering treatments (Table 1).

The first treatment, C, involved olive trees grown under rainfed conditions. The first deficit irrigation treatment T_1_, or the producer’s practice treatment, refers to the method of adding water based on the years of producer experience in grove irrigation and visual inspection of the grove without precisely determined regimes. Second deficit irrigation treatment T_2_ (SAN-technology) refers to reduced irrigation according to phenological stages (Table 2): 80% of ETc before flowering, fruit growth and oil accumulation in the fruit and 50% of ETc between these stages. T_3_ refers to the irrigation treatment involving compensating 100% of the water lost by evapotranspiration (ET_c_) during the entire irrigation period to calculate the amount of water saved using other treatments.

Water was supplied in all irrigation treatments by drip irrigation, with 29 self-compensating drippers placed for each tree around the canopy (flow rate of 2.8 L/h). The amount of water used in all treatments was measured by flow rate meters F0550 (Conrad Electronic SE, Wels, Austria).

The amount of water used in each treatment (IR) was determined using the following Formula (1) [31]:

(1) IR=ETc−EP−R

where ETc is evapotranspiration, EP is effective precipitation and R is available water. Effective precipitation (EP) used in the calculation was 70% of the total precipitation recommendation for olive trees grown in the Mediterranean [31].

Evapotranspiration (ETc) was calculated using the Formula (2) [31,32]:

(2) ETc=ETo×Kc

where ETo is reference evapotranspiration obtained from the PinovaMeteo meteorological station [23] and Kc is corrective factor for olive trees [32]. The Kc value for the months of March, April and May was 0.76, for June it was 0.70, for July and August 0.63, for September 0.72, for October 0.77 and for November 0.75 [32].

Available water (R) used in the calculation was obtained using the following Equation (3):

(3) R=0.70×AWC

where AWC is available water capacity obtained using the following formula (4):

(4) $$\mathrm{AWC}=\frac{\mathrm{PC}-\mathrm{PWC}}{100\times \mathrm{Dr}}$$

where PC refers to field water capacity (% volume), PWC refers to permanent wilting point (% volume), and Dr refers to the depth at which the majority of root system is located.

### 2.3. Harvest of Olive Fruits and Virgin Olive Oil Production

Healthy olive fruits from Coratina cv. olive trees were harvested manually during the second half of October 2019 and 2020 (three trees per treatment) in similar ripening stages (ripening index, RI = 1.5–2.0) determined by the method of Beltrán et al. [33]. The fruits collected from each tree were processed within 24 h of harvesting into oil, separately, to obtain three oil samples per treatment. Oil samples were obtained by processing olive fruits (3 kg per repetition) by centrifugal extraction at the Abencor laboratory oil mill (MC2, Ingenierias y Sistems, Seville, Spain). The olives were ground with a metal hammer mill, while mixing was carried out at a temperature of 25 ± 1 °C in thermostatted vertical mixers for 40 min. After mixing, the olive paste was centrifuged for 90 s at a speed of 3500 rpm, and the oil together with the vegetable water was discharged into separation cylinders. The decanted oil was clarified by additional centrifugation using a Hettich Universal 320R (Andreas Hettich GmbH & Co. KG, Tuttlingen, Germany) for 1 min at 4000 rpm after which it was separated from the vegetable water by decantation and used as a sample. All samples were subsequently stored in dark glass bottles during analyses, which started immediately after oil production.

### 2.4. Virgin Olive Oil Analyses

#### 2.4.1. Oil Content and Oil Yield

Theoretical oil content in the fruit (expressed in fresh and dry weight based on the gravimetric determination of water in fruit) was determined from the olive paste obtained after crushing using a Soxtec Avanti 2.055 apparatus (Foss Tecator, Höganäs, Sweden) according to the method described by Brkić et al. [34].

Oil yield (%) was calculated from three parallel processing repetitions, multiplying by 100 the mass ratio of mechanically extracted oil (g) and centrifuged olive paste (g). Oil yield (OY) was obtained using the following formula (5) [35],

(5) $$\mathrm{OY}=\frac{\mathrm{EO}}{\mathrm{OP}}\times 100$$

where EO is mass of mechanically extracted oil and OP is mass of centrifuged olive paste.

#### 2.4.2. Quality parameters of Virgin Olive Oils

Basic quality parameters of virgin olive oils (VOO), free fatty acids (FFA), peroxide value (PV) and spectrophotometric indices (K_232_, K_270_, and ΔK) were determined according to the methods presented in the European Commission Regulation [36].

#### 2.4.3. Analysis of Pigments in Virgin Olive Oils

Chlorophyll and carotenoid concentrations were determined using a Varian Cary 50 UV/Vis spectrophotometer (Varian Inc., Harbour City, CA, USA) following the procedure of Mínguez-Mosquera et al. [37] and expressed as pheophytin *a* and lutein content (mg/kg), respectively. Briefly, a sample of oil (7.5 g) was weighed and dissolved in cyclohexane in a 25 mL flask. Chlorophyll and carotenoid concentrations were calculated from the oil absorption spectrum. Absorption at 670 nm is associated with the chlorophyll fraction, whose main component is pheophytin a. The pigment that has the highest concentration in the carotenoid fraction is lutein, and its absorption was measured at 470 nm. The values of the specific extinction coefficients used to calculate the pigment concentrations were E0 = 613 for pheophytin and E0 = 2000 for lutein. The following formulas was used to calculate chlorophyll (6) and for carotenoid (7) concentration:

(6) $$\mathrm{Chlorophyll}=\frac{A_{670}\times 10^{6}}{613\times 100\times d}$$

where A_670_ is absorbance on 670 nm, d is thickness of spectrophotometric cuvette, 613 is value of coefficient of specific extinction for phephitin *a* (E_0_);

(7) $$\mathrm{Carotenoid}=\frac{A_{470}\times 10^{6}}{2000\times 100\times d}$$

where A_470_ is absorbance on 470 nm, d is thickness spectrophotometric cuvette, 2000 is value of coefficient of specific extinction for lutein (E_0_).

#### 2.4.4. Analysis of Fatty Acid Methyl Esters (FAME)

Fatty acid methyl esters (FAME) analysis was performed according to the European Commission Regulation [36] using a Varian 3.350 GC (Varian Inc., Harbour City, CA, USA) equipped with an Rtx-2.330 capillary column (105 m length, × 0.25 mm i.d. × 0.25 µm f.t.; Restek, Bellefonte, PA, USA) and a flame-ionization detector. Identification of FAMEs in oil samples was based on their retention times with respect to the standard FAME mixture (Sigma, Darmstadt, Germany) according to the reference method [36]. Relative amounts were expressed as proportions (%) of total fatty acids.

#### 2.4.5. Analysis of Volatile Compounds

The volatile compounds were extracted by the headspace solid-phase microextraction (HS-SPME) according to the method reported by Brkić Bubola et al. [38]. The SPME fibre divinylbenzene/Carbox-en/polydimethylsiloxane (DVB/CAR/PDMS), 1 cm length, 50/30 µm f.t. (Supelco, Bellefonte, PA, USA) was used. The volatile compounds analysis was performed according to the method reported by Brkić Bubola et al. [38] using a Varian 3350 gas chromatograph (Varian Inc., Harbor City, CA, USA) equipped with a split/ splitless injector operating at 245 °C, a flame ionisation detector (FID) operating at 248 °C, and a capillary column Rtx-WAX (60 m length × 0.25 mm i.d. × 0.25 µm f.t.; Restek, Bellefonte, PA, USA). The carrier gas was helium at 138 kPa at the column head. Identification of volatile compounds was performed using a Varian 3900 GC coupled to a Varian Saturn 2100 T ion trap mass spectrometer (Varian Inc., Crawley, UK), by comparing their retention times and mass spectra with those of pure standards and with mass spectra from the NIST05 library. Quantification was carried out using calibration curves of pure standards. For other compounds, semi-quantitative analysis was carried out, and their concentrations (mg/kg) were expressed as equivalents of the compounds with a similar chemical structure for which standards were available, assuming a response factor equal to one [38]. The total volatiles was reported as the sum of all identified volatile compounds.

#### 2.4.6. Analysis of Phenolic Compounds

Extraction and HPLC analysis of phenolic compounds using an Agilent Infinity 1.260 System (Agilent Technologies, Santa Clara, CA, USA) in oil samples was performed according to the methods proposed by Jerman Klen et al. [39] and slightly modified by Lukić et al. [40]. The analysis was accomplished by using an Agilent Infinity 1260 System (Agilent Technologies, Santa Clara, CA, USA) equipped with a G1311B quaternary pump, G1329B autosampler, G1316A column oven, and G4212B DAD detector. A Kinetex PFP column (100 mm length × 4.6 mm i.d., 2.6 µm particle size) with a guard (2.1 mm length × 4.6 mm i.d.; Phenomenex, Sydney, Australia) was used. The flow rate of eluents was 1 mL/min in a 20-step gradient run [40]. Identification of peaks was performed by comparing retention times and UV/Vis spectra with those of pure standards and those from the literature [39]. The detection was carried out at 280 nm for simple phenols, lignans, secoiridoids and vanillic acid, at 320 nm for vanillin and *p*-coumaric acid, and at 365 nm for flavonoids. For quantification, standard calibration curves were made for tyrosol, hydroxytyrosol, vanillic acid, vanillin, *p*-coumaric acid, luteolin, apigenin, pinoresinol and oleuropein. Based on constructed calibration curves, concentrations of samples were expressed as mg/kg oil. Semiquantitative analysis was performed for hydroxytyrosol acetate, acetoxypinoresinol and secoiridoids, where the concentration was expressed as hydroxytyrosol, pinoresinol and oleuropein, respectively, assuming a response factor equal to one. Total phenolic content was presented as the sum of all the identified phenolic compounds.

#### 2.4.7. Radical-Scavenging Activity Determination

Antioxidant capacity of extra virgin olive oil (EVOO) was estimated by evaluating the free radical-scavenging effect of DPPH radical, following the method described by Koprivnjak et al. [41]. Ethyl acetate was used as a solvent to prepare 1 mL of olive oil solution (0.05 g/mL) and 4 mL of DPPH radical solution (0.1 mmol/L). The solutions were vortexed together for 10 s. DPPH• in ethyl acetate was used as a blank solution. Absorbance was measured after 30 min of incubation at 515 nm with a recording interval of 1 min on a Varian Carry 50 spectrophotometer (Varian Inc., Mulgrave, Victoria, Australia). Calibration curves were obtained using Trolox solutions of known concentration (0.0–4.0 mmol/L). The results were presented as mmol (Trolox equivalent)/kg oil according to the calibration curve equation.

#### 2.4.8. Sensory Analysis

Quantitative descriptive sensory analysis of VOO samples was performed by the panel for sensory assessment of VOO, accredited for VOO sensory analysis according to the EN ISO/IEC 17025:2007 and recognized in continuation by the International Olive Council (IOC) from 2014. The panel consisted of eight assessors (5 female, 3 male, average age 33) trained for VOO sensory analysis according to the IOC method [42]. Odour and taste attributes were quantified using a 10-cm unstructured intensity ordinal rating scale from 0 (no perception) to 10 (the highest intensity). Additionally, a modified evaluation sheet expanded with particular odour and taste attributes (green grass/leaves, apple, almond, aromatic herbs, chicory/rocket, green almond peel and astringent) was utilised by the panel to better explain particular changes of sensorial profiles of investigated monovarietal oils.

#### 2.4.9. Data Elaboration

To investigate the effects of different watering regimes of olive trees on the VOO’s parameters, results of the chemical and sensorial analysis were subjected to a one-way analysis of variance (ANOVA). Means were compared by the Tukey’s honest significant difference test at the level of *p* ≤ 0.05. Statistical analysis was carried out using Statistica v. 13.2 software (Stat-Soft Inc., Tulsa, OK, USA).

## 3. Results and Discussion

### 3.1. Influence on Oil Content and Oil Yield

Irrigation was associated with an increase of oil content (on dry- and on the fresh-weight basis) in both years of investigation but there was no significant difference among applied watering regimes, while the percentage of water and dry matter in olive paste did not change (Table 3).

Hernández et al. [43] reported that oil content in the olive fruit related to the deficit irrigation treatments (30% and 60% of full irrigation) was lower than in the full irrigation treatment of Arbequina cultivar during the olive fruit development, while after fruit maturation, no significant difference among different irrigation treatments were determined. Increase of irrigation induces changes in the chemical composition of the olive fruit typical for an advanced maturation stage [18] and higher accumulation of oil in the fruits could be one of them. Consequently, an increase in oil content in the olive fruit influenced an increase in the oil yield obtained (Table 3). In 2020, the increase was the highest in the T_3_ treatment (full irrigation), while in 2019 there was no significant difference among irrigated treatments (Table 3). Pierantozzi et al. [44] also did not find that water availability influenced oil yield in the case of spring deficit irrigation (25–75% ET_c_) compared to fully irrigated Genovese cv. olive trees. Likewise, Moriana et al. [45] did not find differences among rainfed, RDI, 100%, and 125% ET_c_ in oil yield in a study conducted in low density olive orchards in Spain.

Even all applied irrigation strategies have shown great influence on oil yield, considered as economically the most important parameter of olive oil production, in T_1_ and T_2_ treatments, significant savings of water needed for irrigation were observed compared to full irrigation (Table 1).

### 3.2. Influence on VOOs Quality and Composition

#### 3.2.1. Quality Parameters

According to oil basic quality parameters (FFA, PV, K_232_, K_270_, ΔK), all investigated samples were in the range established for the EVOO quality category (Table 4).

In respect of FFA, a quality parameter related to hydrolytic deterioration of oil, only a slight decrease was noted in the T_3_ sample from 2019. Irrigation had a positive effect on PV value, a parameter related to the primary oxidation of oil, and a slight decrease of PV was detected in both years in all irrigated treatments. However, this observation was especially marked in the T_2_ and T_3_ treatments. For the parameters connected to the secondary oxidation of oil, irrigation had no effect on K_232_, while a converse effect on K_270_ values was determined in 2019 and 2020.

Caruso et al. [16] found that quality parameters of olive oil did not allow identifying a clear pattern of variation in respect of soil water availability. FFA was not affected by irrigation of Frantoio cv. trees, while PV and K_232_ values decreased with increase of water deficit but only in one year of the three year investigation [16]. Conversely, Faghim et al. [9] reported the higher value of FFA in irrigated Chemlali oil samples compared to rainfed samples. Tovar et al. [46] did not find any effect of irrigation on FFA or spectrophotometric indices of Arbequina olive oils during a three year study period. Several studies also reported negligible or no effect on oil quality parameters (FFA, PV, K_232_, K_270_) with respect to irrigation [15,47,48,49].

#### 3.2.2. Pigments

Oil pigments, chlorophyll and carotenoids, influence the colour of olive oil and play an important role in oil oxidative stability [50]. However, these are not one of the basic quality parameters of oils included in EU standards [36].

**Figure 2:**
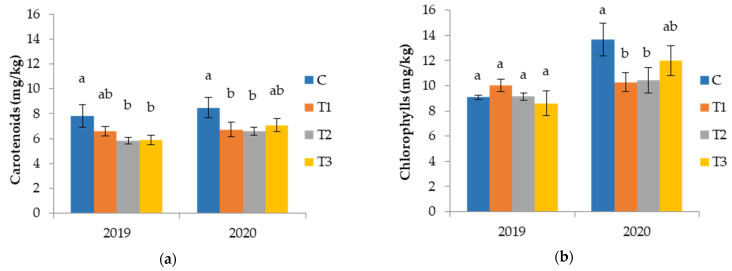
Pigment content: (**a**) carotenoids and (**b**) chlorophyll of virgin olive oils from Coratina cv. olive trees grown under different watering regimes during two crop seasons. Results are expressed as mean values ± standard deviation of three independent repetitions. Mean values labeled with different lowercase letter within the same harvesting year are statistically different (Tukey’s test, *p* ˂ 0.05). Watering regimes: C—rainfed conditions; T_1_—deficit irrigation (the usual producer’s practice); T_2_—deficit irrigation acquired by SAN technology in respect to phenological stages; T_3_—irrigation with 100% of evapotranspiration (ETc) level.

Results from our study illustrated in Figure 2a showed that carotenoid content decreased in oils obtained from irrigated trees. Decrease of carotenoids content after irrigation has been reported by other authors [9,51]. Differences among irrigation treatments were not found during our investigation period of two years (Figure 2b). Similarly, Tovar et al. [46] found no clear difference among irrigation treatments (100%, 70%, 50%, 25% of full irrigation) for oil pigments across three different seasons. Thus, water reduction in summer has no effect on pigments in the olives at harvest time and consequently the content of pigments in the obtained oils.

The effect of irrigation on chlorophyll content was different across our two year period of investigation. In 2020, irrigation was noted to result in a decrease in chlorophyll content with no differences among irrigation treatments; however, in 2019, irrigation did not affect the content of chlorophyll in the olive oils (Figure 2b).

Fernandez et al. [18] reported that chlorophyll contents extracted from the less-watered olive fruits were higher compared to those from the more-watered fruits, concluding that several factors could influence chlorophyll content, such as the effectiveness of the chloroplast breakage during the grinding of the fruits as well as the level of activity of the enzymes (chlorophylases, peroxidases, lipoxygenases) which are influenced by irrigation and could destroy chlorophyll in the oil. Contradictory results regarding chlorophyll content after irrigation have also been found in the literature. For example, Faghim et al. [9] reported a decrease in chlorophyll content from irrigated Chemlali oils comparing to rainfed samples, while other authors reported increases in chlorophyll after irrigation [51,52]. Sdiri et al. [49] did not find differences in chlorophyll in oil from rainfed treatment and waste water irrigated treatments (20 and 40% ETc) of Chemlali olive trees in Tunisia.

#### 3.2.3. Fatty Acid Methyl Esters

In Table 5 the effect of different watering regimes on the fatty acid profile in Coratina olive oils is shown. The fatty acid composition of oils produced from fruits of Coratina cv. irrigated as well as rainfed trees covered ranges expected for EVOO according to EU regulation [36].

Irrigation had a different influence on saturated fatty acids (SFA) and monounsaturated fatty acids (MUFA) in the two years of investigation. In 2019, SFA slightly decreased (due to decrease of palmitic acid), while MUFA content increased (mostly due to an increase of oleic acid content) in all irrigated treatments regardless level of water applied, while in 2020 the influence of irrigation was the opposite. However, these changes in fatty acid composition were very slight, and have no high nutritional relevance.

In the literature, different effect of irrigation on fatty acid composition has been reported. Sdiri et al. [49] have not found differences in fatty acid composition in oil from rainfed treatment and waste water irrigated treatments (20 and 40% ETc) of Chemlali olive trees grown in Tunisia. Fragepane et al. [15] reported that rainfed Cornicabra olive oils had a slightly higher content of oleic acid than irrigated ones, and Faghim et al. [9] also found that irrigation decreases the level of oleic acid in Chemlali oils compared to rainfed samples.

In our study during both years of investigation, polyunsaturated fatty acid (PUFA) content decreased (due to the decrease in linolenic acid content) in oils from irrigated treatments (Table 5). Linoleic acid content decreased in all irrigation treatments when compared to the rainfed treatment, with no significant difference among different irrigation regimes found. These results are consistent with Hernández et al. [43] under conditions of 30%, 60% and 100% of full irrigation of Arbequina olive trees. Other authors have also found a reduction of linoleic acid content in deficit irrigation strategies [16,53]. On the other hand, Caruso et al. [16] found that linoleic acid content in oils increases by increasing the water volume used for irrigated Frantoio olive trees in two of the three years of their investigation. Caruso et al. [16] hypothesized that water stress has a slight effect on fatty acid composition in relatively cool climate conditions, while in hot areas or years, an increase in water likewise increases the linoleic acid with a decrease in oleic acid noted.

Hernández et al. [43] concluded that the effect of irrigation on fatty acids composition depends on cultivar and meteorological conditions. Tovar et al. [46] also concluded that crop season had a greater influence on fatty acid composition of Arbequina cv. oil than irrigation treatments during their three-year study. The fatty acid composition of olive oil was slightly affected by irrigation of Frantoio cv. trees, but changes were dependent on the growing season and clear response of fatty acids composition to different level of irrigation were not determined [16].

In respect of the oleic/linoleic acid ratio, a more favorable ratio (˃7; more ratio value means that oil is more stable to oxidation) was determined in irrigated treatments in both years of our study (Table 5). This was also confirmed by quality parameters related to primary oil oxidation (PV) (Table 4). The highest oleic/linoleic ratio was determined in the T_1_ treatment in 2019, while in 2020, no significant difference among irrigated treatments was found. Higher oleic/linoleic acid ratios in oils obtained from irrigated olives has been reported by other authors [9,43,51].

#### 3.2.4. Volatile Compounds

In Table 6, the volatile profile of VOOs obtained from Coratina cv. olive fruits harvested from rainfed olive trees and irrigated trees at different watering regimes is shown.

During both years of investigation, irrigation was associated with a significant decrease in total volatile compounds, especially in particular groups of volatiles such as total C6 volatiles, as well as total aldehydes and alcohols, which are mostly involved in the specific green odor of VOOs. Regarding the concentration of the most abundant volatile compound, *E*-2-hexenal (related to the green sensory notes), it followed the trend of total volatile compounds. Moreover, in 2019 the decrease of *E*-2-hexanal and total volatile compounds was most pronounced in T_2_ and T_3_ treatments, while in 2020 differences among irrigated treatments were not found. Total ketones were not affected by irrigation in 2019, while in 2020 a decrease was detected in the T_2_ and T_3_ treatments. Total esters increased in all irrigated treatments in 2019, while in 2020 differences among treatments were not found.

Fernandes-Silva et al. [54] also reported that total volatiles tend to decrease with the amount of water applied (rainfed, deficit irrigation 30% ETc, and full irrigation treatments) in the study of irrigation of Cobrancosa cv. trees in Portugal. However, that effect was more pronounced in a year with severe drought than in a year with a rainy spring, thus suggesting that the tree water status influenced the volatile composition of oils not only in the oil accumulation phase but also throughout the whole crop season.

In contrast, Sdiri et al. [49] did not find differences in most of the investigated volatile compounds concentration between oil from rainfed and waste water irrigated treatments (20 and 40% ETc) of Chemlali olive trees grown in Tunisia, except for a slight decrease in octanal and acetic acid concentration in irrigated treatments.

Even though there have been reports that soil water availability could have an influence on the VOO volatile composition [16,55], it seems that the effect of irrigation is cultivar dependent [17]. Dabbou et al. [17] found that total volatiles increased as the amount of supplied water decreased in Arbequina oils, while they increased in Coratina oils and for Koroneiki oils the highest volatile content was observed with a restitution of 75% Et_c_. Therefore, Dabbou et al. [17] concluded that there was no general trend in total volatile changes due to the irrigation regime applied.

From Table 6 it is evident that growing season influenced the concentration of volatile compounds, with generally higher concentration of volatiles in 2020. Although, according to the meteorological station, 2020 had less precipitation overall compared to 2019 [23], higher precipitation in the ripening period in 2020 (Figure 1) could have led to the higher concentration of volatiles determined in that year. Caruso et al. [16] concluded also during their three year investigation of Frantoio cv. tree irrigation that the changes of volatile compounds concentration in olive oils were more dependent on growing season conditions than irrigation regimes. Despite that, Caruso et al. [16] found that some volatiles could be related to the water status of olive trees; *E*-2-hexen-1-al was higher in fully irrigated trees than in watering stressed trees, and higher concentration of aldehydes were found in the irrigated Frantoio cv. trees (full and deficit irrigation), which does not align with our results on volatiles in irrigated Coratina cv. oils.

#### 3.2.5. Phenolic Compounds and Antioxidant Capacity

Table 7 shows the profile of phenolic compounds in VOOs obtained from olive fruits harvested from rainfed olive trees and irrigated at different watering regimes. Coratina oils, produced from the olive fruits harvested in 2019 and in 2020 from the trees grown on the ameliorate karst soil in Croatia, were characterized by very high concentrations of phenolic compounds (more than 1000 mg/kg). In both years of our study, watering regimes applied did not significantly affect the concentration of total phenolic compounds (TPC) determined as well as the concentration of secoiridoids. The lack of difference between T_3_ (100% ETc treatment) and deficit irrigation treatments (T_1_, T_2_) in TPC indicated that similar content could be achieved with less demand in the water supply.

Sdiri et al. [49] also did not find differences in TPC or phenolic profiles between oil from rainfed treatment and oil from waste water irrigated treatments (20 and 40% ETc) of Chemlali olive trees grown in Tunisia. However, Moriana et al. [45] found different effects of irrigation in two consecutive years: in one-year an increased water value during irrigation decreased the TPC value, while in the second year of investigation there were no significant differences found among rainfed, RDI, 100% and 125% ETc treatments for TPC levels. The effect of irrigation on TPC in oil could depend on irrigation levels but also on the specific cultivar [45,47]. Nevertheless, the reduction of TPC levels was mostly reported in irrigated treatments compared to the rainfed conditions [9,15,54,55]. Caruso et al. [47] reported that oils from Frantoio cv. olive trees with a higher water status showed a lower concentration of phenolic compounds. Higher concentration of phenolic content in rainfed oil samples could be due to plant stress responses and defence mechanisms due to severe conditions involving water deficit [9]. Water deficit could activate the phenylalanine ammonia lyase (PAL) enzyme involved in the polyphenols biosynthetic pathway, which could be one of the reasons for the increase in concentration of some particular polyphenols in olive oils in response to water stress during drought [9,46]. In both years of our investigation, there were no severe drought conditions in September, just before fruit harvesting (Figure 1) and that is probably one of the reasons for no significant difference among watering treatments in TPC and secoiridoids. The rain event prior to harvesting increases the fruit water content, which reduce dissolution of phenolic compounds in the oil and have marked impact on olive oil phenolic content [18].

Regarding the concentration of secoiridoids, contradictory data can be found in the literature. Faghim et al. [9] have found a slight increase in some important secoiridoids (ligstrostride aglycon) in olive oil after irrigation, compared with oils obtained from rainfed Chemlali trees. On the contrary, most authors [15,16,56] have found that the level of secoiridoids diminished as the water level during irrigation increased. Fregapane et al. [15] reported the highest level of secoiridoids in rainfed olive oils in two consecutive crop seasons.

Conversely, the concentration of simple phenols increased in all irrigated treatments, especially in the T_3_ treatment, due to increased concentration of hydroxytyrosol and tyrosol (Table 7). However, Faghim et al. [9] reported that these phenolic alcohols were negatively affected by the irrigation of Chemlali olive trees in Tunisia. Caruso et al. [16] found that concentrations of tyrosol were similar for all irrigation treatments (full, deficit, complementary) in Frantoio olive oil, while changes of hydroxytyrosol due to irrigation regimes applied were not the same in each of three years of investigation.

Phenolic acids decreased in treatments with irrigation in 2019, especially in T_3_, while in 2020 that decrease was not significant (Table 7). Faghim et al. [9] reported different influences of irrigation on particular phenolic acids, where o-cumaric acid decreased, while caffeic and ferulic acids increased with irrigation.

The concentration of lignans and flavonoids was not affected by irrigation in 2019, while a slight decrease was detected in 2020 in all irrigated treatments, regardless of the amount of water used for irrigation (Table 7). The concentration of lignans was also not affected by irrigation regimes (full, deficit, complementary) in Frantoio olive oil [16]. Faghim et al. [9] have found a slight increase in flavonoids in olive oils after irrigation, compared with oils obtained from rainfed Chemlali trees.

**Figure 3:**
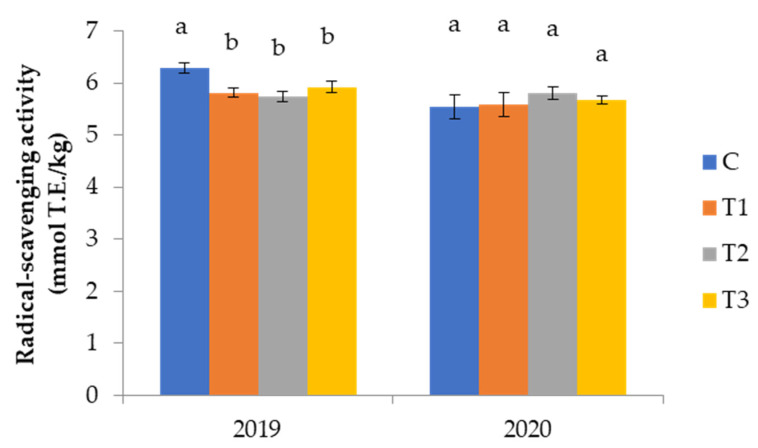
Radical-scavenging activity of virgin olive oils from Coratina cv. olive trees grown under different watering regimes during two crop seasons: (**a**) 2019 and (**b**) 2020. Watering regimes: C—rainfed conditions; T_1—_deficit irrigation (the usual producer’s practice); T_2_—deficit irrigation acquired by SAN technology in respect to phenological stages; T_3_—irrigation with 100% of evapotranspiration (ETc) level. Results are expressed as mean values ± standard deviation of three independent repetitions. Mean values labeled with different lowercase letter within the same characteristic and within the same harvesting year are statistically different (Tukey’s test, *p* ˂ 0.05).

Irrigation caused a slight decrease in the antioxidant capacity of VOO in 2019 regardless of which irrigation regimes were used. On the other hand, in 2020, the effect of irrigation was not confirmed since there was no difference found among all the treatments (Figure 3), supporting the results of total phenolic compounds determined in corresponding oils (Table 7).

#### 3.2.6. Sensory Characteristics

**Figure 4:**
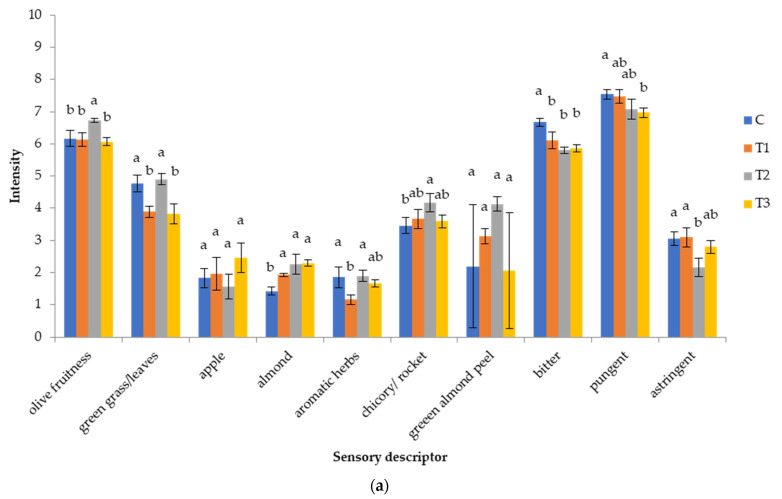

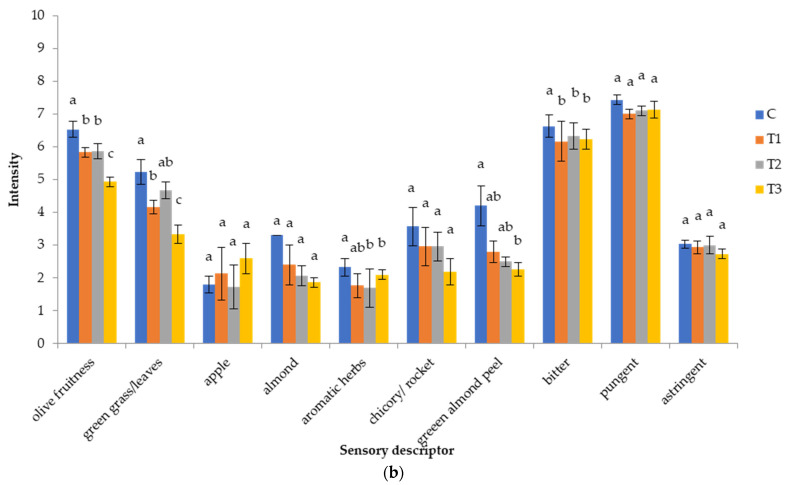
Sensory characteristics of virgin olive oils from Coratina cv. olive trees grown under different watering regimes during two crop seasons: (**a**) 2019 and (**b**) 2020. Watering regimes: C—rainfed conditions; T_1_—deficit irrigation (the usual producer’s practice); T_2_—deficit irrigation acquired by SAN technology in respect to phenological stages; T_3_—irrigation with 100% of evapotranspiration (ETc) level. Results are expressed as mean values ± standard deviation of three independent repetitions. Mean values labeled with different lowercase letter within the same characteristic and within the same harvesting year are statistically different (Tukey’s test, *p* ˂ 0.05).

According to the results of sensory analysis (Figure 4), all investigated VOO could be considered as extra virgin olive oil (EVOO) since any defect did not occur and the fruitiness of the samples were detected [42].

Moreover, intensities of fruitiness, bitterness and pungency of Coratina cv. oils from rainfed trees grown on karst soil in Croatia were robust (intensities more than 6). Regarding the particular aroma descriptors, in 2019 the highest intensity of fruitiness was determined in the T_2_ treatment, and notes of green grass and leaves decreased in T_1_ and T_3_ treatment compared to control rainfed oil, while in T_2_ these were similar to the control (C) oil obtained from rainfed fruits. In 2020, irrigation influenced a decrease in fruitiness and green aroma (green grass and leaves, radicchio) of obtained oils, especially in the case of T_3_. Decrease in green aroma corresponded with a decrease of *E*-2-hexenal and C6 volatiles determined in oils from irrigated treatments (Table 6). Dabbou et al. [17] found that cultivars responded differently to the irrigation regimes: the fruitiness attribute increased in the Koroneiki cultivar as an increase in the irrigation level occurred, while an opposite trend was observed in Arbeqina oils. Fragepane et al. [15] found that Cornicabra oil fruitiness was not affected by irrigation in one year of their investigation, while in the other year of investigation, fruitiness decreased slightly with an increasd in the water delivered through irrigation.

Considering the taste characteristics of olive oils, in 2019 bitterness and pungency of oil only slightly decreased in all treatments with irrigation, while in 2020, even though a decrease was found, it was not significant. Differences in taste characteristics among irrigated treatments were not detected (Figure 4). Dabbou et al. [17] found that bitter and pungent attributes were more evident in oils from less irrigated treatments. Fernandes-Silva et al. [54] found that sensory characteristics, pungent and bitter, were more pronounced in the Cobrancosa cv. olive oils from rainfed and deficit irrigation (30% ET_c_) treatments than in full irrigation treatments (100% ET_c_). Fragepane at al. [15] found that bitterness and pungency were not affected by irrigation in one year of their study, while in the other year bitterness decreased slightly because of an increase in water delivered through irrigation. Fragepane et al. [15] suggested that a decrease in bitterness for Cornicabra oil could positively affect consumer opinion about olive oil since a high level of Cornicabra oil bitterness could lead to rejection of the oil. Since Coratina oils also had high intensity of bitterness, detected decrease of bitterness in irrigated treatments could have positive effects on consumer acceptance of this oil.

## 4. Conclusions

In this study, we examined the influence of different watering regimes applied to Coratina cv. trees grown on ameliorate karst soils in Croatia, with respect to oil quantity, composition and quality. Olive trees were subjected to rainfed conditions and three different irrigation treatments (T_1_—deficit irrigation representing the usual producer’s practice, T_2_—regulated deficit irrigation in respect to phenological stages, T_3_—full irrigation) during two years of investigation.

Our results indicated that Coratina cv. olive trees grown on the karst soil in Croatia could produce EVOO of high quality under rainfed conditions, as well as under irrigated conditions, while under irrigation conditions, the quantity of obtained oil is higher.

Carotenoids content decreased in oils obtained from irrigated fruits, but differences among different irrigation treatments were not determined in both years of investigation. On the other hand, the irrigation effect on chlorophyll content was contradictory across the two years of investigation. PUFA and linolenic acid contents decreased in all irrigation treatments compared to rainfed treatment, with no significant difference among different irrigation regimes. In general, irrigation influenced a decrease in volatile compounds involved in the specific green odor of VOOs. Watering regimes applied did not significantly affect the concentration of total phenolic compounds and secoiridoids determined, while the concentration of simple phenols increased in all irrigated treatments. Intensities of fruitiness, bitterness and pungency of Coratina cv. oils from rainfed trees grown on karst soil in Croatia were robust and the detected decrease of bitterness in irrigated treatments could have positive effects on consumer acceptance of this oil.

No difference between T_3_ (100% ETc treatment) and deficit irrigation treatments (T_1_, T_2_) in oil composition indicated that similar VOO quality could be achieved with less demand in water supply. Our results also indicated that T_2_, “smart agriculture” treatment, although it had not produced statistically different results in quantity and quality of oil in regard to other irrigation regimes investigated, enabled precise and automatic water rate application, thus greatly increasing water usage efficiency while saving energy and human labor. Additionally, this strategy resulted in sustainable water saving due to a reduction of water needed for irrigation between critical phenological stages (flowering, pit hardening and oil accumulation). The obtained results could help producers to define a suitable irrigation management scheme in the particular condition of ameliorate karst in Croatia to obtain olive oil of high quality but also to produce a greater quantity of oil. Since the year of harvest could have great influence on the olive oil composition, continued investigation of irrigation in the karst soil conditions in Croatia during more harvesting years is needed.

## Figures and Tables

**Table 1:** Amount of water added per tree, number of rates and the amount of water saved as compared to T_3_ over the two-year period (2019 and 2020).

Treatment *	2019	2020	2019	2020	2019	2020
	Amount (l)		Rate Number		Saved Water (l)	
C	0	0	0	0	800	1800
T_1_	448	1393	5	11	352	407
T_2_	560	1261	8	19	240	539
T_3_	800	1800	8	19	0	0

* Watering regimes: C—rainfed conditions; T_1_—deficit irrigation (the usual producer’s practice); T_2_—deficit irrigation acquired by SAN technology in respect to phenological stages; T_3_—irrigation with 100% of evapotranspiration (ETc) level.

**Table 2:** Dates of phenological stages in olive cv. Coratina during 2019 and 2020.

Phenological Stages	BBCH * [30]	2019	2020
Flowering	61–68	3/5–15/5	2/5–12/5
Fruit set	69	16/5–20/5	13/5–19/5
Pit hardening	75	10/7–23/7	11/7–20/7
Oil accumulation	79–89	10/8–10/9	10/8–10/9

* BBCH—Biologische Bundesanstalt, Bundessortenamt und Chemische Industrie.

**Table 3:** Content of moisture, dry matter, oil on dry and fresh weight basis in olive paste, and oil yield during the production of virgin olive oils from Coratina cv. olive trees grown under different watering regimes during two crop seasons (2019 and 2020).

Parameter	2019				2020			
	C	T_1_	T_2_	T_3_	C	T_1_	T_2_	T_3_
Oil yield (%)	5.55 ± 0.76 ^b^	8.83 ± 0.50 ^a^	9.09 ± 0.20 ^a^	9.31 ± 0.95 ^a^	5.58 ± 0.32 ^c^	8.69 ± 0.21 ^b^	9.44 ± 0.55 ^ab^	10.11 ± 0.50 ^a^
Dry matter (%)	44.79 ± 1.87 ^a^	47.14 ± 0.75 ^a^	47.16 ± 0.45 ^a^	46.96 ± 2.12 ^a^	42.91 ± 1.00 ^a^	43.96 ± 1.11 ^a^	45.00 ± 0.40 ^a^	44.66 ± 0.63 ^a^
Moisture (%)	55.21 ± 1.87 ^a^	52.86 ± 0.75 ^a^	52.84 ± 0.45 ^a^	53.04 ± 2.12 ^a^	57.09 ± 1.00 ^a^	56.04 ± 1.11 ^a^	55.00 ± 0.40 ^a^	55.34 ± 0.63 ^a^
Oil on dry weight basis (%)	21.29 ± 0.74 ^b^	28.04 ± 0.95 ^a^	29.69 ± 2.01 ^a^	30.98 ± 0.93 ^a^	27.16 ± 1.87 ^b^	37.54 ± 1.44 ^a^	36.65 ± 1.79 ^a^	38.87 ± 0.70 ^a^
Oil on fresh weight basis (%)	11.76 ± 0.74 ^b^	14.83 ± 0.62 ^a^	15.69 ± 1.17 ^a^	16.42 ± 0.52 ^a^	15.51 ± 1.18 ^b^	21.04 ± 1.12 ^a^	20.16 ± 1.04 ^a^	21.51 ± 0.22 ^a^

Results are expressed as mean values ± standard deviation of three independent repetitions. Mean values labeled with different lowercase superscript letter within the same row and within the same harvesting year are statistically different (Tukey’s test, *p* ˂ 0.05). Watering regimes: C—rainfed conditions; T_1_—deficit irrigation (the usual producer’s practice); T_2_–deficit irrigation acquired by SAN technology in respect to phenological stages; T_3_—irrigation with 100% of evapotranspiration (ETc) level.

**Table 4:** Quality parameters (peroxide value—PV; free fatty acids—FFA; spectrophotometric indices—K_232_, K_270_ and ∆K) of virgin olive oils from Coratina cv. olive trees grown under different watering regimes during two crop seasons (2019 and 2020).

	2019					2020			
	C	T_1_	T_2_	T_3_	C	T_1_	T_2_	T_3_	EVOO *
FFA % (oleic acid)	0.20 ± 0.00 ^a^	0.19 ± 0.01 ^ab^	0.18 ± 0.01 ^ab^	0.17 ± 0.01 ^b^	0.19 ± 0.00 ^a^	0.19 ± 0.01 ^a^	0.18 ± 0.01 ^a^	0.18 ± 0.01 ^a^	≤2.50
PV (meq O_2_/kg)	2.34 ± 0.05 ^a^	1.89 ± 0.02 ^b^	1.63 ± 0.05 ^c^	1.68 ± 0.08 ^c^	2.21 ± 0.06 ^a^	1.56 ± 0.03 ^b^	1.19 ± 0.11 ^c^	1.06 ± 0.06 ^c^	≤20.0
K_232_	1.94 ± 0.07 ^a^	1.93 ± 0.12 ^a^	1.82 ± 0.10 ^a^	1.97 ± 0.07 ^a^	1.91 ± 0.06 ^a^	2.02 ± 0.05 ^a^	2.04 ± 0.02 ^a^	2.02 ± 0.07 ^a^	≤0.22
K_270_	0.17 ± 0.01 ^a^	0.15 ± 0.02 ^b^	0.15 ± 0.00 ^ab^	0.15 ± 0.01 ^ab^	0.17 ± 0.00 ^b^	0.19 ± 0.01 ^ab^	0.20 ± 0.00 ^a^	0.19 ± 0.01 ^ab^	≤0.01
∆K	0.00 ± 0.01 ^a^	0.00 ± 0.00 ^a^	0.00 ± 0.00 ^a^	0.00 ± 0.00 ^a^	0.00 ± 0.01 ^a^	0.00 ± 0.00 ^a^	0.00 ± 0.00 ^a^	0.00 ± 0.00 ^a^	

Results are expressed as mean values ± standard deviation of three independent repetitions. Mean values labeled with different lowercase superscript letter within the same row and within the same harvesting year are statistically different (Tukey’s test, *p* ˂ 0.05). * Actual limits for extra virgin olive oil (EVOO) category [36]. Watering regimes: C—rainfed conditions; T_1_—deficit irrigation (the usual producer’s practice); T_2_—deficit irrigation acquired by SAN technology in respect to phenological stages; T_3_—irrigation with 100% of evapotranspiration (ETc) level.

**Table 5:** Fatty acid profile (%) of virgin olive oils from Coratina cv. olive trees grown under different watering regimes during two crop seasons (2019 and 2020).

	2019				2020				
	C	T_1_	T_2_	T_3_	C	T_1_	T_2_	T_3_	EVOO *
Myristic (C 14:0)	0.01 ± 0.00 ^a^	0.01 ± 0.00 ^a^	0.01 ± 0.00 ^a^	0.01 ± 0.00 ^a^	0.01 ± 0.00 ^a^	0.01 ± 0.00 ^a^	0.01 ± 0.00 ^a^	0.01 ± 0.00 ^a^	≤0.03
Palmitic (C 16:0)	13.72 ± 0.30 ^a^	12.32 ± 0.11 ^b^	12.10 ± 0.23 ^b^	12.39 ± 0.13 ^b^	10.30 ± 0.35 ^c^	12.28 ± 0.18 ^b^	12.77 ± 0.23 ^b^	13.62 ± 0.29 ^a^	7.50–20.00
Palmitoleic (C 16:1)	0.95 ± 0.09 ^a^	0.60 ± 0.02 ^b^	0.62 ± 0.03 ^b^	0.65 ± 0.04 ^b^	0.85 ± 0.03 ^a^	0.64 ± 0.04 ^b^	0.60 ± 0.02 ^b^	0.58 ± 0.02 ^b^	0.30–3.50
Heptadecanoic (C 17:0)	0.08 ± 0.01 ^a^	0.04 ± 0.00 ^b^	0.03 ± 0.00 ^b^	0.04 ± 0.01 ^b^	0.05 ± 0.00 ^a^	0.05 ± 0.01 ^a^	0.05 ± 0.00 ^a^	0.05 ± 0.00 ^a^	≤0.40
Heptadecenoic (C 17:1)	0.07 ± 0.01 ^a^	0.07 ± 0.00 ^a^	0.07 ± 0.00 ^a^	0.07 ± 0.00 ^a^	0.07 ± 0.00 ^a^	0.07 ± 0.01 ^a^	0.07 ± 0.00 ^a^	0.07 ± 0.00 ^b^	≤0.60
Stearic (C 18:0)	2.52 ± 0.04 ^a^	2.61 ± 0.11 ^a^	2.60 ± 0.02 ^a^	2.53 ± 0.14 ^a^	2.39 ± 0.05 ^a^	2.43 ± 0.02 ^a^	2.48 ± 0.01 ^a^	2.40 ± 0.15 ^a^	0.50–5.00
Oleic (C 18:1)	73.40 ± 0.30 ^b^	76.02 ± 0.19 ^a^	75.76 ± 0.14 ^a^	75.55 ± 0.21 ^a^	77.48 ± 0.30 ^a^	76.71 ± 0.30 ^b^	76.18 ± 0.16 ^b^	75.56 ± 0.11 ^c^	55.00–83.00
Linoleic (C 18:2)	7.30 ± 0.12 ^a^	6.55 ± 0.20 ^b^	7.06 ± 0.10 ^a^	7.06 ± 0.07 ^a^	6.84 ± 0.10 ^a^	6.14 ± 0.04 ^b^	6.18 ± 0.04 ^b^	6.13 ± 0.04 ^b^	2.50–21.00
Linolenic (C18:3)	1.00 ± 0.03 ^a^	0.81 ± 0.02 ^b^	0.77 ± 0.01 ^b^	0.78 ± 0.02 ^b^	0.95 ± 0.03 ^a^	0.71 ± 0.03 ^b^	0.68 ± 0.02 ^b^	0.65 ± 0.05 ^b^	≤1.00
Arachidic (C 20:0)	0.41 ± 0.02 ^a^	0.43 ± 0.01 ^a^	0.43 ± 0.00 ^a^	0.41 ± 0.03 ^a^	0.43 ± 0.01 ^a^	0.41 ± 0.00 ^a^	0.42 ± 0.00 ^a^	0.40 ± 0.03 ^a^	≤0.60
Eicosenoic (C 20:1)	0.36 ± 0.01 ^a^	0.38 ± 0.01 ^a^	0.38 ± 0.01 ^a^	0.37 ± 0.01 ^a^	0.44 ± 0.02 ^a^	0.39 ± 0.01 ^ab^	0.40 ± 0.00 ^ab^	0.38 ± 0.04 ^b^	≤0.50
Behenic (C 22:0)	0.11 ± 0.03 ^a^	0.11 ± 0.01 ^a^	0.11 ± 0.00 ^a^	0.10 ± 0.01 ^a^	0.12 ± 0.00 ^a^	0.11 ± 0.00 ^b^	0.11 ± 0.00 ^b^	0.10 ± 0.01 ^b^	≤0.20
Eicosenoic acid (C 22:1)	n.d.	n.d.	n.d.	n.d.	n.d.	n.d.	n.d.	n.d.	
Lignoceric (C 24:0)	0.05 ± 0.01 ^a^	0.06 ± 0.00 ^a^	0.05 ± 0.01 ^a^	0.05 ± 0.00 ^a^	0.05 ± 0.00 ^a^	0.05 ± 0.00 ^a^	0.05 ± 0.00 ^a^	0.05 ± 0.00 ^a^	≤0.20
18:2t + 18:3t	n.d.	n.d.	n.d.	n.d.	n.d.	n.d.	n.d.	n.d.	≤0.05
∑ SFA	16.90 ± 0.28 ^a^	15.57 ± 0.05 ^b^	15.32 ± 0.23 ^b^	15.53 ± 0.15 ^b^	13.35 ± 0.31 ^c^	15.33 ± 0.20 ^b^	15.89 ± 0.23 ^b^	16.63 ± 0.12 ^a^	
∑ MUFA	74.79 ± 0.39 ^b^	77.07 ± 0.20 ^a^	76.84 ± 0.13 ^a^	76.63 ± 0.20 ^a^	78.85 ± 0.28 ^a^	77.82 ± 0.25 ^b^	77.25 ± 0.18 ^b^	76.58 ± 0.10 ^b^	
∑ PUFA	8.31 ± 0.12 ^a^	7.35 ± 0.20 ^c^	7.84 ± 0.10 ^b^	7.84 ± 0.08 ^b^	7.79 ± 0.12 ^a^	6.85 ± 0.06 ^b^	6.86 ± 0.06 ^b^	6.78 ± 0.09 ^b^	
Oleic/linoleic ratio (C18:1/C18:2)	10.05 ± 0.20 ^c^	11.62 ± 0.38 ^a^	10.73 ± 0.13 ^b^	10.70 ± 0.13 ^b^	11.33 ± 0.17 ^b^	12.49 ± 0.12 ^a^	12.33 ± 0.06 ^a^	12.32 ± 0.10 ^a^	

Results are expressed as mean values ± standard deviation of three independent repetitions. Mean values labeled with different lowercase superscript letter within the same row and within the same harvesting year are statistically different (Tukey’s test, *p* ˂ 0.05). 18:2t + 18:3t—Total *trans* linoleic and *trans* linolenic isomers; SFA—saturated fatty acids; MUFA—monounsaturated fatty acids; PUFA—polyunsaturated fatty acids; n.d.—not determined. * Actual limits for extra virgin olive oil (EVOO) category [36]. Watering regimes: C—rainfed conditions; T_1_—deficit irrigation (the usual producer’s practice); T_2_—deficit irrigation acquired by SAN technology in respect to phenological stages; T_3_—irrigation with 100% of evapotranspiration (ETc) level.

**Table 6:** Volatile compounds concentration of virgin olive oils from Coratina cv. olive trees grown under different watering regimes during two crop seasons (2019 and 2020).

Volatile Compounds (mg/kg)	2019				2020			
	C	T_1_	T_2_	T_3_	C	T_1_	T_2_	T_3_
3-methylbutanal	0.95 ± 0.06 ^a^	0.63 ± 0.07 ^b^	0.65 ± 0.01 ^b^	0.73 ± 0.04 ^b^	0.04 ± 0.01 ^a^	0.03 ± 0.00 ^a^	0.03 ± 0.00 ^a^	0.03 ± 0.01 ^a^
3-pentanone	0.07 ± 0.00 ^b^	0.08 ± 0.00 ^ab^	0.08 ± 0.00 ^a^	0.08 ± 0.00 ^a^	0.09 ± 0.01 ^a^	0.09 ± 0.02 ^a^	0.07 ± 0.00 ^b^	0.05 ± 0.00 ^b^
1-penten-3-one	0.80 ± 0.04 ^a^	0.84 ± 0.03 ^a^	0.84 ± 0.02 ^a^	0.84 ± 0.04 ^a^	3.22 ± 0.46 ^a^	2.94 ± 0.30 ^a^	2.10 ± 0.11 ^b^	1.78 ± 0.13 ^b^
Ethyl 2-methylbutanoate	0.93 ± 0.03 ^b^	1.23 ± 0.00 ^a^	1.24 ± 0.02 ^a^	1.21 ± 0.07 ^a^	n.d.	n.d.	n.d.	n.d.
Hexanal	6.00 ± 0.18 ^a^	6.23 ± 0.10 ^a^	6.36 ± 0.34 ^a^	5.95 ± 0.19 ^a^	2.10 ± 0.31 ^a^	1.45 ± 0.10 ^b^	1.50 ± 0.01 ^b^	1.10 ± 0.06 ^b^
(*Z*)-2-pentenal *	0.08 ± 0.01 ^a^	0.09 ± 0.01 ^a^	0.09 ± 0.00 ^a^	0.09 ± 0.01 ^a^	0.03 ± 0.01 ^b^	0.06 ± 0.01 ^a^	0.05 ± 0.01 ^a^	0.05 ± 0.01 ^a^
Isoamyl acetate	0.10 ± 0.01 ^b^	0.12 ± 0.01 ^a^	0.11 ± 0.01 ^ab^	0.11 ± 0.01 ^ab^	n.d.	n.d.	n.d.	n.d.
(*E*)-2-pentenal	0.13 ± 0.01 ^a^	0.13 ± 0.00 ^a^	0.12 ± 0.01 ^a^	0.11 ± 0.01 ^a^	0.16 ± 0.01 ^a^	0.13 ± 0.00 ^b^	0.09 ± 0.00 ^c^	0.08 ± 0.00 ^d^
(*E*)-3-hexenal *	0.87 ± 0.02 ^ab^	0.98 ± 0.06 ^a^	0.73 ± 0.08 ^b^	0.95 ± 0.08 ^a^	0.25 ± 0.03 ^b^	0.31 ± 0.01 ^a^	0.25 ± 0.01 ^b^	0.19 ± 0.02 ^c^
(*Z*)-3-hexenal *	0.04 ± 0.00 ^a^	0.04 ± 0.00 ^a^	0.03 ± 0.00 ^a^	0.03 ± 0.01 ^a^	0.60 ± 0.06 ^b^	1.63 ± 0.19 ^a^	1.95 ± 0.26 ^a^	1.61 ± 0.12 ^a^
(*Z*)-2-hexenal *	0.22 ± 0.01 ^a^	0.22 ± 0.01 ^b^	0.17 ± 0.02 ^b^	0.19 ± 0.01 ^ab^	0.98 ± 0.06 ^a^	0.73 ± 0.03 ^b^	0.64 ± 0.02 ^b^	0.68 ± 0.03 ^b^
(*E*)-2-hexenal	16.48 ± 0.5 ^a^	12.79 ± 0.09 ^b^	11.29 ± 0.32 ^c^	11.47 ± 0.16 ^c^	85.07 ± 9.42 ^a^	49.13 ± 8.15 ^b^	42.71 ± 1.98 ^b^	36.34 ± 1.85 ^b^
Hexyl acetate	0.03 ± 0.00 ^ab^	0.03 ± 0.00 ^a^	0.03 ± 0.00 ^ab^	0.02 ± 0.00 ^b^	0.08 ± 0.00 ^a^	0.08 ± 0.00 ^a^	0.07 ± 0.00 ^a^	0.07 ± 0.00 ^a^
Octanal	0.04 ± 0.00 ^a^	0.03 ± 0.00 ^b^	0.03 ± 0.00 ^ab^	0.04 ± 0.00 ^c^	0.28 ± 0.01 ^a^	0.26 ± 0.00 ^b^	0.28 ± 0.00 ^a^	0.26 ± 0.00 ^b^
(*E*)-2-penten-1-ol	0.47 ± 0.03 ^bc^	0.62 ± 0.07 ^a^	0.58 ± 0.01 ^ab^	0.43 ± 0.02 ^c^	0.16 ± 0.01 ^a^	0.14 ± 0.01 ^b^	0.11 ± 0.00 ^c^	0.09 ± 0.00 ^d^
(*Z*)-2-penten-1-ol + (*Z*)-3-hexenyl acetate	0.75 ± 0.02 ^a^	0.75 ± 0.03 ^ab^	0.68 ± 0.03 ^b^	0.69 ± 0.03 ^ab^	4.69 ± 0.37 ^a^	4.29 ± 0.3 ^a^	3.39 ± 0.21 ^b^	2.58 ± 0.18 ^c^
Hexanol	0.05 ± 0.00 ^a^	0.04 ± 0.01 ^b^	0.03 ± 0.00 ^b^	0.03 ± 0.00 ^b^	0.03 ± 0.00 ^a^	0.02 ± 0.00 ^b^	0.02 ± 0.00 ^b^	0.02 ± 0.00 ^b^
(*E*)-3-hexen-1-ol	n.d.	n.d.	n.d.	n.d.	n.d.	n.d.	n.d.	n.d.
(*Z*)-3-hexen-1-ol	1.15 ± 0.05 ^a^	0.77 ± 0.02 ^b^	0.44 ± 0.03 ^c^	0.48 ± 0.06 ^c^	0.79 ± 0.11 ^a^	0.36 ± 0.01 ^b^	0.30 ± 0.01 ^b^	0.25 ± 0.02 ^b^
(*E*)-2-hexen-1-ol	0.33 ± 0.01 ^a^	0.29 ± 0.00 ^ab^	0.23 ± 0.05 ^b^	0.24 ± 0.02 ^b^	0.47 ± 0.01 ^a^	0.29 ± 0.07 ^b^	0.34 ± 0.01 ^b^	0.28 ± 0.03 ^b^
(*Z*)-2-hexen-1-ol	0.25 ± 0.02 ^a^	0.07 ± 0.00 ^b^	0.05 ± 0.00 ^b^	0.05 ± 0.00 ^b^	n.d.	n.d.	n.d.	n.d.
(*E*)-2-octenal	1.48 ± 0.06 ^a^	0.82 ± 0.02 ^b^	0.83 ± 0.04 ^b^	0.77 ± 0.03 ^b^	n.d.	n.d.	n.d.	n.d.
Total C5 volatiles	1.75 ± 0.04 ^a^	1.79 ± 0.02 ^a^	1.73 ± 0.05 ^a^	1.72 ± 0.07 ^a^	3.65 ± 0.48 ^a^	3.35 ± 0.31 ^a^	2.43 ± 0.12 ^b^	2.05 ± 0.13 ^b^
Total C6 volatiles	24.05 ± 0.53 ^a^	20.14 ± 0.12 ^b^	18.38 ± 0.47 ^c^	18.2 ± 0.30 ^c^	90.36 ± 9.64 ^a^	54.00 ± 8.22 ^b^	47.78 ± 1.8 ^b^	40.54 ± 2.00 ^b^
Total aldehydes	25.05 ± 0.60 ^a^	20.6 ± 0.08 ^b^	19.25 ± 0.55 ^c^	19.03 ± 0.33 ^c^	89.19 ± 9.56 ^a^	53.41 ± 8.19 ^b^	47.17 ± 1.80 ^b^	40.02 ± 1.98 ^b^
Total alcoholes	2.29 ± 0.07 ^a^	1.84 ± 0.05 ^b^	1.38 ± 0.10 ^c^	1.44 ± 0.03 ^c^	1.28 ± 0.12 ^a^	0.67 ± 0.06 ^b^	0.66 ± 0.01 ^b^	0.55 ± 0.05 ^b^
Total esters	0.96 ± 0.03 ^b^	1.26 ± 0.01 ^a^	1.27 ± 0.02 ^a^	1.24 ± 0.07 ^a^	0.08 ± 0.00 ^a^	0.08 ± 0.00 ^a^	0.07 ± 0.00 ^a^	0.07 ± 0.00 ^a^
Total ketones	0.87 ± 0.05 ^a^	0.91 ± 0.03 ^a^	0.93 ± 0.02 ^a^	0.92 ± 0.04 ^a^	3.31 ± 0.47 ^a^	3.02 ± 0.32 ^a^	2.17 ± 0.11 ^b^	1.83 ± 0.13 ^b^
Total volatile compounds	31.24 ± 0.54 ^a^	26.79 ± 0.24 ^b^	24.61 ± 0.41 ^c^	24.51 ±0.49 ^c^	99.01 ± 9.25 ^a^	61.94 ± 8.28 ^b^	53.91 ± 2.12 ^b^	45.46 ± 2.20 ^b^

Results are expressed as mean values ± standard deviation of three independent repetitions. Mean values labeled with different lowercase superscript letter within the same row and within the same harvesting year are statistically different (Tukey’s test, *p* ˂ 0.05). Watering regimes: C—rainfed conditions; T_1_—deficit irrigation (the usual producer’s practice); T_2_—deficit irrigation acquired by SAN technology in respect to phenological stages; T_3_—irrigation with 100% of evapotranspiration (ETc) level. N.d.—not determined. * The volatile compounds for which pure standards were not available were quantified semi-quantitatively, and their concentrations (mg/kg) were expressed as equivalents of the compounds with similar chemical structure for which standards were available, assuming a response factor = 1.

**Table 7:** Concentration of phenolic compounds of virgin olive oils from Coratina cv. olive trees grown under different watering regimes during two crop seasons (2019 and 2020).

Phenolic Compounds (mg/kg)	2019				2020			
	C	T_1_	T_2_	T_3_	C	T_1_	T_2_	T_3_
Simple phenols								
Tyrosol	6.0 ± 1.0 ^c^	8.1 ± 0.4 ^cb^	9.9 ± 1.2 ^ab^	12.3 ± 0.8 ^a^	5.7 ± 0.5 ^d^	9.0 ± 0.9 ^c^	11.5 ± 0.9 ^b^	13.8 ± 1.0 ^a^
Hydroxytyrosol	4.1 ± 0.7 ^d^	6.0 ± 0.5 ^c^	8.4 ± 1.0 ^b^	10.3 ± 0.6 ^a^	2.4 ± 0.2 ^c^	4.5 ± 0.8 ^b^	6.2 ± 0.4 ^a^	7.7 ± 0.8 ^a^
Hydroxytyrosol acetate *	0.1 ± 0.0 ^a^	0.1 ± 0.0 ^a^	0.1 ± 0.0 ^a^	0.1 ± 0.0 ^a^	0.1 ± 0.0 ^a^	0.1 ± 0.0 ^b^	0.1 ± 0.0 ^c^	0.1 ± 0.0 ^bc^
Vanillin	0.1 ± 0.0 ^b^	0.2 ± 0.0 ^a^	0.2 ± 0.0 ^ab^	0.1 ± 0.0 ^ab^	0.2 ± 0.0 ^a^	0.2 ± 0.0 ^b^	0.1 ± 0.0 ^b^	0.1 ± 0.0 ^b^
Total simple phenols	10.3 ± 1.8 ^c^	14.4 ± 0.08 ^bc^	18.5 ± 2.2 ^b^	22.8 ± 1.1 ^a^	8.4 ± 0.3 ^d^	13.8 ± 1.7 ^c^	17.9 ± 1.25 ^b^	21.7 ± 1.7 ^a^
Phenolic acids								
Vanillic acid	0.2 ± 0.0 ^a^	0.2 ± 0.0 ^a^	0.2 ± 0.2 ^ab^	0.1 ± 0.1 ^b^	2.9 ± 0.8 ^a^	2.7 ± 0.6 a	2.6 ± 0.2 ^a^	2.5 ± 0.4 ^a^
*p*-Coumaric acid	1.2 ± 0.2 ^a^	1.2 ± 0.1 ^a^	1.2 ± 0.1 ^a^	1.0 ± 0.1 ^a^	2.2 ± 0.2 ^a^	1.6 ± 0.1 ^b^	1.5 ± 0.1 ^b^	1.5 ± 0.1 ^b^
Total phenolic acids	1.5 ± 0.2 ^a^	1.4 ± 0.1 ^ab^	1.34 ± 0.1 ^ab^	1.17 ± 0.1 ^b^	5.1 ± 0.9 ^a^	4.3 ± 0.6 ^a^	4.0 ± 0.1 ^a^	4.0 ± 0.4 ^a^
Flavonoids								
Luteolin	0.8 ± 0.2 ^a^	1.1 ± 0.3 ^a^	0.9 ± 0.1 a	1.0 ± 0.2 ^a^	1.8 ± 0.2 ^a^	1.5 ± 0.1 ^ab^	1.2 ± 0.2 ^b^	1.3 ± 0.2 ^ab^
Apigenin	0.1 ± 0.0 ^a^	0.1 ± 0.0 ^a^	0.1 ± 0.0 ^a^	0.1 ± 0.0 ^a^	0.3 ± 0.0 ^a^	0.2 ± 0.0 ^ab^	0.2 ± 0.0 ^b^	0.2 ± 0.0 ^b^
Total flavonoids	0.9 ± 0.2 ^a^	1.2± 0.4 ^a^	1.0 ± 0.1 ^a^	1.1 ± 0.2 ^a^	2.0 ± 0.2 ^a^	1.7 ± 0.1 ^ab^	1.4 ± 0.2 ^b^	1.5 ± 0.2 ^b^
Lignans								
Pinoresinol	3.2 ± 0.1 ^c^	3.7 ± 0.0 ^cb^	4.3 ± 0.1 ^ab^	4.8 ± 0.6 ^a^	4.5 ± 0.4 ^a^	3.1 ± 0.3 ^b^	3.9 ± 0.5 ^ab^	4.4 ± 0.4 ^a^
Acetoxypinoresinol *	19.0 ± 2.5 ^a^	19.2 ± 2.3 ^a^	17.3 ± 0.9 ^a^	17.9 ± 3.0 ^a^	20.0 ± 0.4 ^a^	15.4 ± 0.2 ^b^	16.0 ± 1.7 ^b^	16.9 ± 1.6 ^b^
Total lignans	22.1 ± 2.44 ^a^	22.8 ± 2.3 ^a^	21.6 ± 1.0 ^a^	22.8 ± 3.4 ^a^	24.5 ± 0.3 ^a^	18.5 ± 0.1 ^b^	20.0 ± 2.1 ^b^	21.3 ± 1.9 ^ab^
Secoiridoids								
3,4-DHPEA-EDA *	228.8 ± 45.1 ^a^	165.1 ± 7.7 ^a^	181.9 ± 9.4 ^a^	185.1 ± 43.0 ^a^	256.8 ± 34.4 ^a^	133.4 ± 16.4 ^b^	163.3 ± 14.4 ^b^	158.8 ± 26.4 ^b^
Oleuropein aglycone (isomer I) *	449.7 ± 56.4 ^a^	374.6 ± 50.8 ^a^	357.6 ± 35.3 ^a^	348.2 ± 30.1 ^a^	339.2 ± 48.1 ^a^	394.7 ± 38.3 ^a^	382.1 ± 41.2 ^a^	367.7 ± 30.9 ^a^
*p*-HPEA-EDA *	195.6 ± 32.2 ^a^	139.4 ± 17.2 ^a^	159.0 ± 18.7 ^a^	155.0 ± 42.5 ^a^	207.6 ± 18.2 ^a^	181.1 ± 10.3 ^a^	211.6 ± 20.1 ^a^	200.1 ± 9.6 ^a^
Oleuropein + ligstroside aglycones I & II *	258.3 ± 24.9 ^a^	194.6 ± 19.5 ^b^	202.7 ± 23.2 ^ab^	199.6 ± 26.7 ^ab^	186.8 ± 29.5 ^b^	298.4 ± 34.8 ^a^	298.0 ± 29.5 ^a^	284.1 ± 29.4 ^a^
Oleuropein aglycone (isomer II) *	77.8 ± 4.5 ^a^	68.4 ± 5.8 ^a^	75.2 ± 7.8 ^a^	71.5 ± 9.3 ^a^	39.5 ± 6.7 ^a^	41.3 ± 2.7 ^a^	46.4 ± 2.7 ^a^	46.5 ± 0.8 ^a^
Ligstroside aglycone (isomer III) *	16.4 ± 0.6 ^a^	17.8 ± 2.5 ^a^	18.4 ± 1.0 ^a^	20.1 ± 3.4 ^a^	11.3 ± 0.8 ^b^	12.2 ± 0.1 ^b^	14.2 ± 1.6 ^ab^	15.5 ± 1.3 ^a^
Oleuropein aglycone (isomer III) *	50.1 ± 3.5 ^a^	43.5 ± 8.2 ^a^	40.5 ± 7.8 ^a^	40.3 ± 7.7 ^a^	16.1 ± 2.6 ^b^	22.0 ± 2.1 ^b^	30.3 ± 2.9 ^a^	30.7 ± 3.1 ^a^
Total secoiridoids	1276.8 ± 151.6 ^a^	1003.3 ± 108.6 ^a^	1035.3 ± 100.6 ^a^	1019.8 ± 148.1 ^a^	1057.4 ± 123.8 ^a^	1083.2 ± 60.0 ^a^	1146.1± 108.0 ^a^	1103.4± 42.7 ^a^
Total phenolic content	1311.8 ± 153.3 ^a^	1043.3 ± 110.8 ^a^	1077.9 ± 102.2 ^a^	1067.7 ± 152.7 ^a^	1097.5 ±123.7 ^a^	1121.5 ± 59.9 ^a^	1189.4 ± 109.6 ^a^	1152.0 ± 45.9 ^a^

Results are expressed as mean values ± standard deviation of three independent repetitions. Mean values labeled with different lowercase superscript letter within the same row and within the same harvesting year are statistically different (Tukey’s test, *p* ˂ 0.05). * The phenolic compounds for which pure standards were not available were quantified semi-quantitatively, and their concentrations were expressed as equivalents of hydroxytyrosol for hydroxytyrosol acetate, oleuropein for secoiridoids, and pinoresinol for acetoxypinoresinol assuming a response factor = 1. Watering regimes: C—rainfed conditions; T_1—_deficit irrigation (the usual producer’s practice); T_2_—deficit irrigation acquired by SAN technology in respect to phenological stages; T_3_—irrigation with 100% of evapotranspiration (ETc) level.
